# Leptomeningeal Carcinomatosis From Carcinoma of Unknown Primary in a Young Patient: A Case Report and a Literature Review

**DOI:** 10.1177/2324709619869380

**Published:** 2019-08-17

**Authors:** Leila Moosavi, Carlos D’Assumpcao, Jonathan Bowen, Arash Heidari, Everardo Cobos

**Affiliations:** 1 Kern Medical Center—UCLA, Bakersfield, CA, USA; 2 Ross University, Miramar, FL, USA

**Keywords:** carcinoma of unknown primary, leptomeningeal metastasis, leptomeningeal carcinomatosis, ovarian mucinous carcinoma, placental alkaline phosphatase, caudal type homeobox 2, cytokeratin

## Abstract

Leptomeningeal carcinomatosis, leptomeningeal meningitis, or, as referred here, leptomeningeal metastasis (LM), is a rare but frequently fatal complication seen in advanced stage of cancer either locally advanced or after a metastasis of a known primary cancer. We present a rare and uncommon case of leptomeningeal metastases from carcinoma of unknown primary. A 32-year-old female was diagnosed with LM; however, no known primary carcinoma was identified after 2 separate biopsies. The first biopsy of the right pre-tracheal lymph node showed poorly differentiated pan-keratin (AE1 and AE3) and placental alkaline phosphatase with the possibility of germ cell origin. Second cytology of cervical lymphadenopathy was remarkable for cytokeratin 7 and 20, placental alkaline phosphatase, and CDX2 suggestive of germ line tumor with both mucinous ovarian and gastrointestinal carcinomas. Unfortunately, the LM progressed rapidly despite multiple cycles of germ cell origin directed systemic and intrathecal chemotherapy, and the patient opted for hospice care without getting a chance to identify the primary source.

## Introduction

Leptomeningeal metastasis (LM) is defined as infiltration of the leptomeninges by malignant cells. The most common solid tumors giving rise to leptomeningeal metastases are reported to be breast, lung, melanoma,^1^ and cancers of unknown primary, which represents only 1% to 7% of all cases.^2^ Patients can present with a wide range of nonspecific signs and symptoms resulting from involvement of various sites in the craniospinal axis. Diagnosis can be challenging and often requires a high index of suspicion by clinicians.

## Case Report

**Figure 1.**
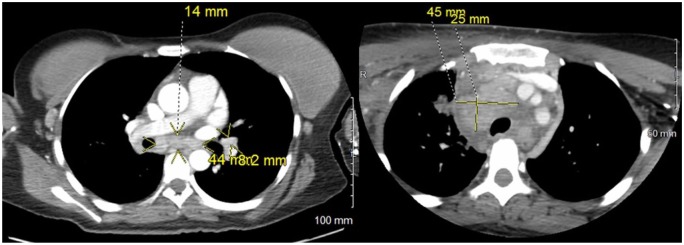
Computed tomography chest angiography to rule out pulmonary embolism instead found carinal, hilar (left), and paratracheal lymphadenopathy (right).

**Figure 2.**
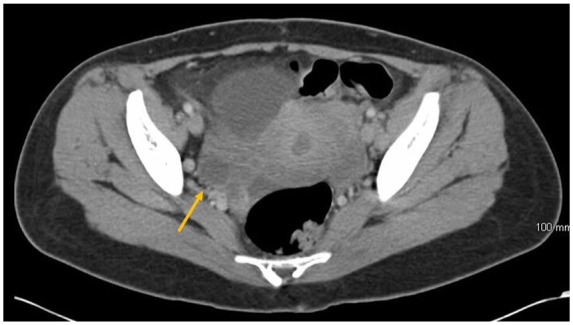
Computed tomography scan of abdomen and pelvis revealed a cystic structure in the right adnexa.

**Figure 3.**
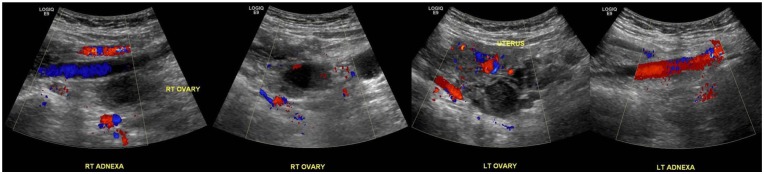
Ultrasound of pelvis did not show any obvious lesions or cystic structures.

A 32-year-old Hispanic female with no comorbidities initially presented to an outside hospital with persistent productive cough, dyspnea, decreased appetite, and unintentional weight loss of 4.5 kg. The patient underwent a computed tomography (CT) angiography to rule out pulmonary embolism, but the imaging found instead moderately prominent intrathoracic lymphadenopathy of uncertain etiology (Figure 1). An infectious workup including for coccidioidomycosis, of which she lives in an endemic area, was negative. CT scan of the abdomen and pelvis demonstrated prominent lymph nodes in the upper retroperitoneal region including a 3.3-cm ovarian cystic structure (Figure 2). However, no discrete lesion or adnexal masses was identified on ultrasound of pelvis (Figure 3). CA 125 was 100 U/mL. And CA 19-9 was 75 U/mL. Lactate acid dehydrogenase was 937 U/L. HE4, AFP, CEA, and βHCG were within normal limits. Given the nonspecific findings on imaging, the patient underwent a diagnostic mediastinoscopy of the right pre-tracheal lymph node. Pathology was suggestive of a poorly differentiated pan-keratin (AE1 and AE3) and placental alkaline phosphatase (PLAP) positive malignant neoplasm. Germ cell tumor was suspected. During the same hospitalization, serum coccidioides immunodiffusion returned immunoglobulin M very weakly reactive; however, immunofixation of complement was less than 1:2. Nonetheless, the patient was started on fluconazole 800 mg daily.

**Figure 4.**
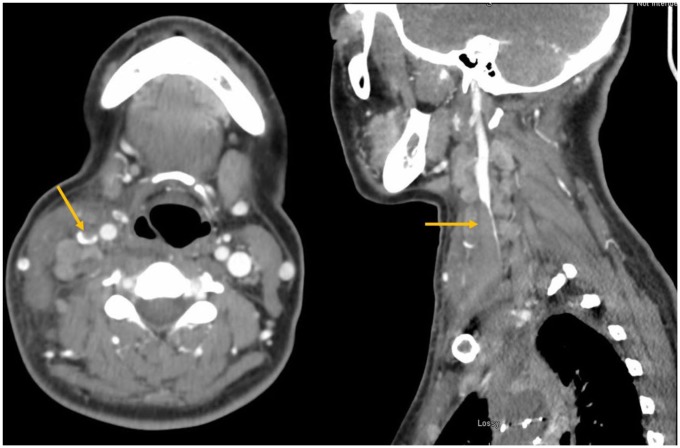
Computed tomography scan of the neck with intravenous contrast revealed thrombus in the right internal jugular vein extending from the mediastinum.

The patient had another hospitalization for neck swelling. Imaging found a thrombus extending from the midportion of the right internal jugular vein down to the superior mediastinum (Figure 4). She was diagnosed with superior vena cava syndrome. She was anticoagulated with rivaroxaban and successfully discharged in stable condition.

As an outpatient, the patient was started on a germ cell origin carcinoma directed chemotherapy regimen of carboplatin and paclitaxel. She, unfortunately, developed an allergic reaction to paclitaxel and it was replaced with docetaxel anhydrous. She subsequently tolerated 3 cycles of carboplatin and docetaxel anhydrous.

**Figure 5.**
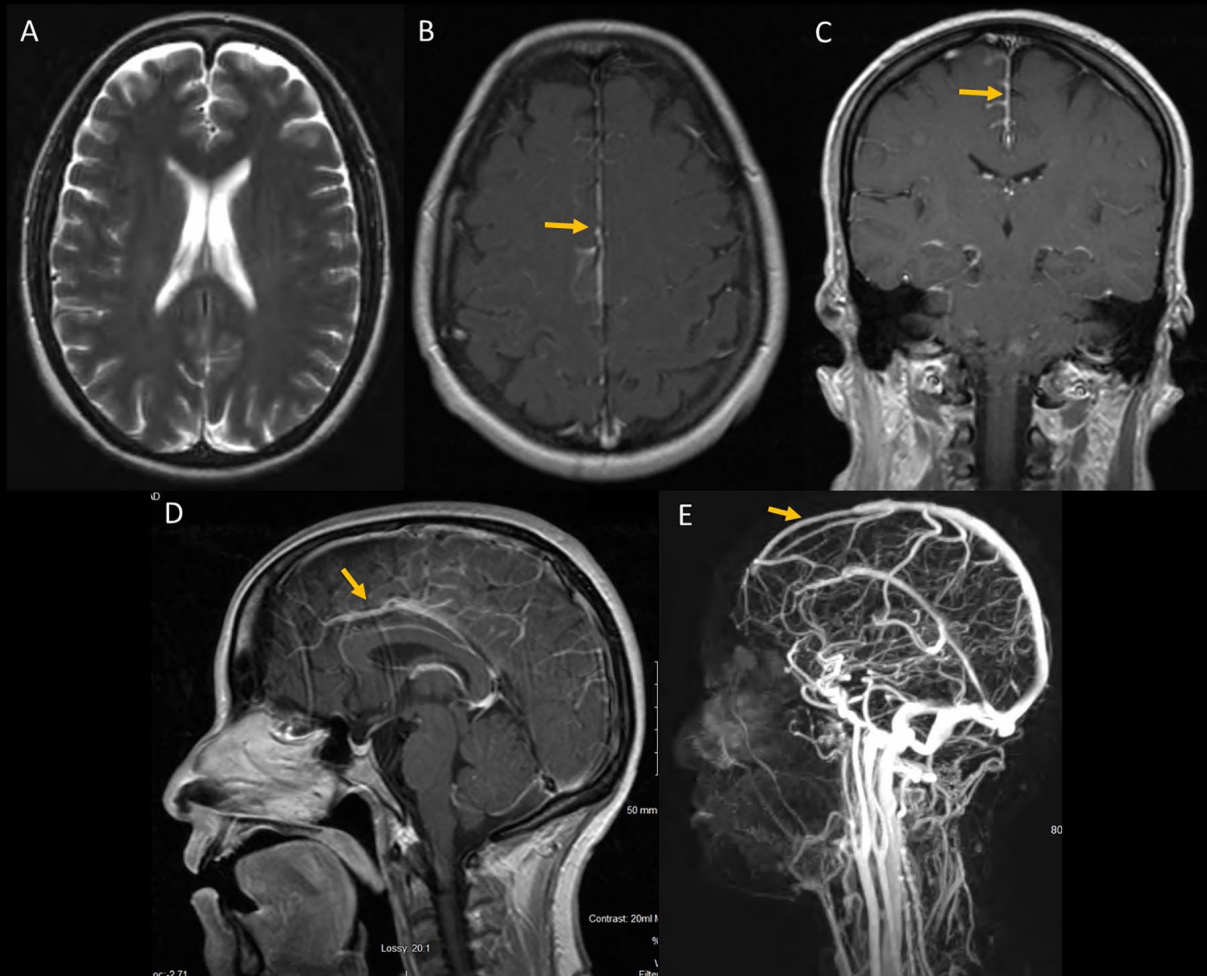
Magnetic resonance imaging of brain T1 axial showed no hydrocephalus (A), leptomeningeal enhancement most prominent in the falx cerebri (seen in axial [B], coronal [C], and sagittal [D] T1 images with gadolinium). Magnetic resonance venography showed a narrowing of the distal cerebral venous sinus (E).

Four months after the initial presentation, the patient presented with 1-week history of headache described as the worst headache of her life. Magnetic resonance venography/magnetic resonance imaging (MRI) were remarkable for slight narrowing of the distal portion of the straight sinus and leptomeningeal enhancement but negative for hydrocephalus (Figure 5).

Patient’s headaches were initially relieved by lumbar punctures (LPs), which had elevated opening pressures. She received serial of LPs for symptomatic relief and diagnostic workups. Broad infectious cerebral spinal fluid investigations were inconclusive. However, cytology found atypical malignant cells, suggestive of leptomeningeal carcinomatosis. It was decided to attempt intrathecal methotrexate (ITMX). Unfortunately, she developed worsening of headaches, photophobia, and neck stiffness with high opening pressures ranging from 30 to 600 cm H_2_O on LPs. The patient was subsequently switched to IT cytarabine twice a week.

**Figure 6.**
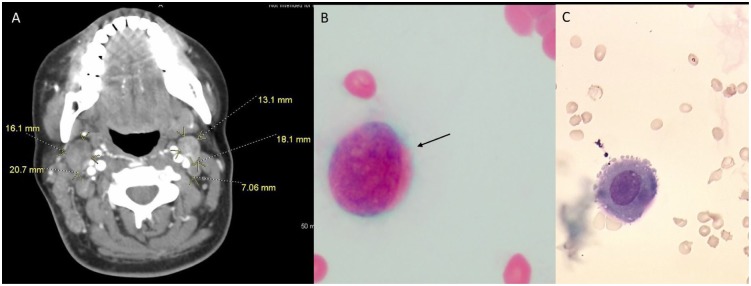
(A) Computed tomography scan of neck found several new lymphadenopathies of increasing sizes. Cerebrospinal fluid (CSF) malignant cells are seen on CSF gram stain (B) and wet mount (C). Placental alkaline phosphatase staining not shown.

**Figure 7.**
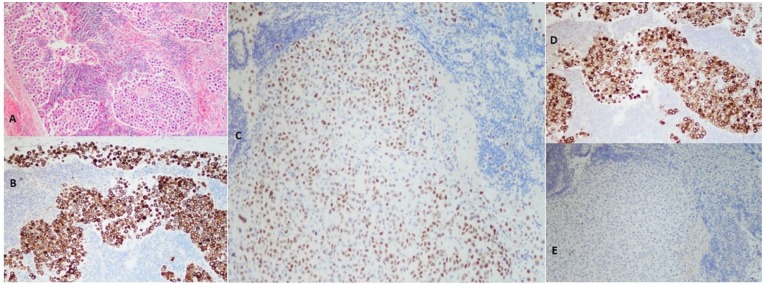
Immunohistochemistry stains of cervical lymph node biopsy. (A) Hematoxylin and eosin, 4×: Showing malignant cells infiltrating subcapsular sinus and spread throughout the sinusoids. (B) CK 7 positive, 10×: seen in lung, breast, and upper gastrointestinal adenocarcinoma type malignancies. (C) CDX2 positive, 10×: Seen in gastrointestinal primary malignancies and ovarian mucinous carcinoma. (D) Placental alkaline phosphatase positive, 10×: Nonspecific, typically associated with germ cell tumor. (E) TTF-1 negative, 10×: Seen in thyroid and lung malignancies.

Search for a primary malignancy began anew. CT scan of chest and neck showed new findings of cervical lymphadenopathy that was not present on previous CT 6 weeks prior (Figure 6). Positron emission tomography scan revealed bulky adenopathy above and below the diaphragm and hypermetabolic pleural-based foci within the bilateral lung fields consistent with malignancy/metastasis. Cervical lymph node biopsy was performed. Immunohistochemical staining and cytopathology focusing on search for occult primary were positive for cytokeratin 7 and 20, PLAP, and CDX2 (Figure 7). Additionally, evidence of PLAP was identified persistently on cerebrospinal fluid (CSF) cytology, which confirms leptomeningeal carcinomatosis (Figure 6).

Despite aggressive intrathecal chemotherapy, she continued to deteriorate with progressively worsening headaches, developed photophobia, and had loss of peripheral vision. Ventricular peritoneal shunt was considered for the elevated intracranial pressures. The patient appeared to be severely deconditioned and continued to have declining performance (Eastern Cooperative Oncology Group performance score of 3 and Karnofsky Performance Status score of 50). After receiving 3 cycles of carboplatin and docetaxel, 1 dose of ITMX, and 4 doses of intrathecal cytarabine, oncology determined that she was a poor candidate to receive additional chemotherapy. In addition, she developed a hematoma at the site of LPs. Altogether, such severe leptomeningeal disease confers a poor prognosis.

After 5 months of fighting her cancer, she refused the palliative intraventricular shunt, additional procedures, and treatment. The patient and her family bravely proceeded with comfort care measures and hospice, 5 weeks after the headache started.

## Discussion

Leptomeningeal metastasis is a devastating complication of almost every known cancer. It has been reported in 5% to 10% of patients with metastatic cancers.^1^ We report an unusual case of LM of unknown primary with features of germ cell carcinoma and gastrointestinal origin from 2 different biopsy sites.

Initially, germ cell tumor primary was suspected (Table 1). The presence of an ovarian cyst, CA-125, LDH, and PLAP highly suggested a form of germ cell tumor; however, negative AFP, βHCG, HE4, and CEA did not allow for further differentiation. Presence of mediastinal lymphadenopathy staged the patient as T × N × M1 or Stage IV. Platinum-based and microtubule-based chemotherapy were chosen to limit the spread of the malignancy. The infusion was tolerable. Unfortunately, development of headache, photophobia with matching CSF, and MRI findings meant leptomeningeal carcinomatosis had developed. Repeat cervical lymph node biopsy was furthermore nonspecific, except for PLAP, which was shared with the initial biopsy. Additionally, cells in the CSF also tested positive for PLAP, further suggesting germ cell tumor metastasis to the meninges. The presence of cytokeratin 7, 20, and CDX2 in the second biopsy of the cervical lymph node, markers used in occult primary malignancy search, suggest remaining tumor cells may have originated from a possible ovarian mucinous carcinoma (Table 1), with CA 125 supporting this finding; however, serum CEA was negative. The strength of PLAP on multiple biopsies and CSF cell staining lend more credence to germ line tumor with some epithelial differentiation into ovarian mucinous carcinoma. Unfortunately, metastasis had already spread to the meninges.

**Table 1.** Summary of Immunohistochemistry Tumor Markers Studied in This Case of Metastasis of Unknown Origin^a^

Tumor Marker	Result	Interpretation
Broad-spectrum cytokeratin (pan-keratin) identified with AE1/AE3 antibody	Positive on second biopsy and cerebrospinal fluid (CSF) cells	Used in cell lineage determination (epithelial vs mesenchymal vs melanocytic). Pan-keratin suggests epithelial differentiation.
Placental alkaline phosphatase (PLAP)	Positive on first and second biopsy and CSF cells	Membrane-bound isoenzyme produced by placental syncytiotrophoblasts and many neoplasms. Sensitive for germ cell tumors such as seminomas and embryonal carcinomas, yolk sac tumors, and choriocarcinomas. Some non–germ cell carcinomas such as serous carcinomas.
Cytokeratin 7	Positive on second biopsy	Because of overlap expression, CK7/20 profiles usually are combined with more specific markers. Cytokeratin 7 and 20 positive tumors include: Urothelial carcinoma (uroplakin, thrombomodulin, p63, CK5/6) Pancreatic ductal adenocarcinoma (CDA, CA19-9, CDX-2) Ovarian mucinous carcinoma (CDK-2) Adenocarcinoma of bladder (thrombomodulin, CDK-2) Gastric adenocarcinoma (subset) Cholangiocarcinoma (subset)
Cytokeratin 20	Focal cells positive on second biopsy	
Caudal type homeobox 2 (CDX-2)	Positive on second biopsy	Nuclear transcription factor expressed in >95% of colorectal and duodenal adenocarcinomas with nuclear staining. Also seen in urachal adenocarcinomas from urinary bladder and ovarian mucinous carcinomas and some neuroendocrine tumors.
Glial fibrillary acidic protein (GFAP)	Negative on CSF cells	Intermediary filament protein important in astrocytes development. Used in glial cell differentiation.^27^
Alpha fetoprotein (AFP)	Negative	Oncofetal glycoprotein in yolk sac tumors and hepatocellular carcinoma.
Beta human chorionic gonadotropin (βHCG)	Negative	Glycoprotein with α and β subunits. β subunit is made in syncytiotrophoblasts. Found in choriocarcinoma, multinucleated syncytiotrophoblastic cells in seminomas, embryonal carcinomas, and yolk sac tumors.
CD30 (tumor necrosis factor receptor family)	Negative	Embryonal carcinomas
c-Kit (CD117)	Negative	Mast/stem cell growth factor receptor (tyrosine protein kinase kit) activated in gastrointestinal stromal tumors.^28^
Melan A	Negative	Melanoma and steroid producing cells in adrenal cortex in adrenal cortical carcinomas.
Gross cystic disease fluid protein (GCDFP-15; BRST-2)	Negative	Prolactin-inducing glycoprotein expressed by apocrine cells of breast, axillary and anogenital skin apocrine glands, salivary glands, and Paget disease of skin; 50% to 74% sensitive and 95% specific for breast primary.
Estrogen receptor (ER)	Negative	Not specific to breast. Cannot be used as evidence of breast primary. May be used in conjunction with GCDFP-15.
GATA3	Negative	One of 6 GATA transcription factors. Involved in luminal differentiation of breast epithelium, development of collecting system/urothelium and trophoblastic differentiation. Regulator of type 2 helper T-cells.^29^
Paired box 8 (PAX8)	Negative	Transcription factor critical to development of eye, thyroid, urinary, and reproductive organs. Associated with tumors of thyroid, kidney/upper urinary tract, and Müllerian system.^30^
Thyroid transcription factor 1 (TTF-1)	Negative	Thyroid adenocarcinoma. Lung adenocarcinoma or small cell lung carcinoma, if thyroid adenocarcinoma is excluded. Also, small cell carcinomas from prostate, urinary bladder, and uterine cervix.

a Pathology results are from an initial mediastinal lymph node, a second biopsy of cervical lymph node as part of search for occult primary, and cerebral spinal fluid cytology. Interpretations are adapted from Krishna^31^ unless otherwise cited. AE1/AE3, PLAP, CK7/20, and CDX-2 positivity suggested a tumor of ovarian/germ cell linage, possibly ovarian mucinous carcinoma. Although CDX-2 is expressed in greater than 95% of colorectal and duodenal adenocarcinomas, these gastrointestinal tumors are classically CK7/20 negative.

There is no guidance for LC with undifferentiated germ cell primary. When primary is unknown, a regimen should be selected to cover breast, lung, and epithelial ovarian cancer for broad coverage. Therefore, ITMX was chosen. Intrathecal cytarabine was also tried after chemical meningitis developed. Unfortunately, chemical meningitis is suspected to have caused extreme pain and headache and increased intracranial pressures without hydrocephalus. Despite multiple cycles of systemic and intrathecal chemotherapy, her disease progressed rapidly and the patient proceeded with hospice care.

The prognosis of LM from known solid tumors is poor, ranging from 4 to 6 weeks without treatment^3^ to 3 to 6 months with treatment.^4^ The primary treatment goal is palliation to improve the neurological deficits as well as the quality of life. Once the diagnosis is made and risk status identified, 3 therapeutic possibilities in LM for those with good risk status and wish to have further therapy. The National Comprehensive Cancer Network (NCCN) provides the following recommendations at a category 2A level of evidence and consensus.^5^ Systemic chemotherapy is specific to primary cancer type, emphasizing drugs with good central nervous system penetration. Intra-CSF chemotherapy can also be considered.^6^ Ommaya or intraventricular catheter placement is recommended. If there are signs and symptoms of CSF flow blockage, it needs to be evaluated with CSF flow scan and treated with fractionated external beam radiotherapy to relieve flow abnormalities, if possible. Whole brain radiation therapy and/or involved-field radiation therapy to bulky disease and neurologically symptomatic (such as cranial neuropathies) or painful sites. Volumes and dose of radiotherapy will depend on primary source and sites requiring palliation.

Chemotherapy options are based on primary malignancy. To date, NCCN recommendations are available for lymphoma, leukemia, breast, non–small cell lung cancers, and occult malignancy. If lymphoma/leukemia primary, additional options are IT cytarabine^7-10^ or ITMX.^9,11^ Lymphomas have additional options in IT rituximab^12^ and systemic methotrexate^13^ as well. If breast cancer is the primary malignancy, ITMX^14,15^ and IT trastuzumab^16^ in addition to systemic methotrexate^17^ can be used. If systemic methotrexate is used in general, consider glucarpidase (carboxypeptidase G2) for prolonged methotrexate clearance due to methotrexate-induced renal toxicity.^18^ If primary cancer is non–small cell lung cancer, options depend on mutation identified. Systemic osimertinib can be used for EGFR mutation-positive tumors.^19^ Systemic weekly pulse erlotinib for EGFR exon 19 deletion or exon 21 L858R mutation can also be used (NCCN category 2B).^5,20^ When a primary malignancy is not identified despite widespread search, occult malignancy is suspected. In these cases, IT N,NI,NII-triethylene phosphoramide (Thio-tepa),^21^ IT topotecan,^22^ IT etoposide,^23^ or IT interferon-α (NCCN category 2B)^5,8^ are recommended.

Evaluation of treatment response depends on clinical status, CSF cytology results, and radiographic status. The goal of therapy is clinical stability or improvement, negative CSF cytology, and no radiologic progression of LM disease. Reevaluation of CSF cytology every 4 to 8 weeks is warranted to assess treatment effectiveness. If treatment is ineffective, switch chemotherapies, radiotherapy to symptom sites, continue systemic chemotherapy, or palliate with best supportive care.

Chemical meningitis is not uncommon with ITMX. Jacob et al^24^ prospectively observed patients undergoing prophylaxis ITMX for acute lymphoblastic leukemia or lymphoblastic lymphoma for signs of post-ITMX syndrome defined as vomiting, headache, and fever. Of 297 doses, 6.7% post-ITMX syndrome were observed.

Chemical meningitis has been also reported with intrathecal liposomal cytarabine.^25^ In this case, prompt identification of chemical meningitis after the first dose in patient with BRAF-V600E-mutated melanoma leptomeningeal metastases allowed for dose delay and reduction and subsequent tolerability of 15 additional injections with appropriate clinical and MRI enhancement response. This particular patient survived to 36 months at least.

## Conclusion

Despite newer targeted immunotherapeutic agents that are currently under investigation, an improved understanding of the etiology of LM, treatment as well as additional randomized trials are needed to be conducted to determine optimal treatment of such a devastating disease.^26^ Nonetheless, LM is still very difficult to treat. Our patient had a very rapid decline.
